# Direct interleukin-6 inhibitor olokizumab in the treatment of polymyalgia rheumatica: a case series

**DOI:** 10.3389/fmed.2026.1735764

**Published:** 2026-05-26

**Authors:** Alexey D. Meshkov, Alexey L. Maslyanskiy, Anton L. Chudinov, Elena P. Ilivanova, Ilya O. Smitienko, Pavel I. Novikov, Anna V. Torgashina, Evgeniy V. Arseniev, Natalia O. Khovasova, Anton V. Naumov, Olga N. Tkacheva, Evgeny G. Zotkin

**Affiliations:** 1 Russian Clinical and Research Center of Gerontology, Moscow, Russia; 2 Almazov National Medical Research Center, Saint Petersburg, Russia; 3 Clinical Rheumatology Hospital No. 25 Named After V.A. Nasonova, Saint-Petersburg, Russia; 4 Leningrad Regional Clinical Hospital, Saint-Petersburg, Russia; 5 Medical Center K+31, Moscow, Russia; 6 University Clinical Hospital #3, Sechenov First Moscow State Medical University, Moscow, Russia; 7 V.A. Nasonova Research Institute of Rheumatology, Moscow, Russia

**Keywords:** corticosteroids, giant cell arteritis, IL-6 inhibition, olokizumab, polymyalgia rheumatica

## Abstract

**Objective:**

To describe the experience of using olokizumab (OKZ) in patients with polymyalgia rheumatica (PMR) who have contraindications or an inadequate response to methotrexate and require steroid-sparing therapy, and to analyze treatment outcomes in this cohort.

**Methods:**

A case series included 23 patients with PMR [including giant-cell arteritis (GCA) in 9 patients] treated with OKZ. The assessment of disease activity was carried out using the PMR-activity score (AS) index, as well as laboratory parameters [erythrocyte sedimentation rate (ESR), C-reactive protein (CRP)]. The statistical analysis was performed using the StatTech v program. 4.7.3.

**Results:**

Olokizumab therapy resulted in a significant decrease in PMR activity: the PMR-AS index decreased from 18.6 (14.2–24.4) to 3.01 (1.62–6.09) points by 12–24th weeks. Most patients (86.3%) who received therapy achieved remission or low disease activity. Laboratory parameters have also improved: ESR values decreased from 32.00 (20.00–47.00) to 7.00 (5.00–8.50) mm/h, and CRP – from 14.10 (8.10–40.80) to 0.85 (0.30–2.15) mg/l. The steroid dose decreased from 7.5 (5.0–15.0) to 2.50 (0.00–2.50) mg/day by week 24. The results listed above were statistically significant (*p* < 0.05). The disease duration of less than 36 months at the time of initiation of therapy proved to be a significant predictor of achieving low PMR activity (AUC = 0.912; *p* = 0.024).

**Conclusion:**

Olokizumab demonstrated high efficacy in reducing the activity of PMR, laboratory markers of inflammation and the dose of steroids in patients with inefficacy or intolerance to methotrexate.

## Introduction

The need for steroid-sparing medication (1) in patients with polymyalgia rheumatica (PMR) and giant cell arteritis (GCA), the two conditions that frequently co-occur, is high. Whereas tocilizumab was approved for GCA a long time ago (2), in spring 2023 the Food and Drug Administration (FDA) approved sarilumab for treatment of PMR, which was the first biologic approval for the management of this disorder (3).

The pivotal SAPHYR study (4), that included 118 patients, confirmed efficacy of sarilumab in patients with PMR. The study assessed the rate of stable remission, which was shown to be significantly higher in sarilumab group, whereas the risk of relapse after achieving clinical remission was 44% lower than in the comparison group. In this trial the chances of reaching remission were almost twice higher, and the cumulative corticosteroid (CS) dose was significantly lower in the sarilumab group. It should be noted that sarilumab, while demonstrating high steroid-free remission rates in clinical trials, has limited availability in the Russian Federation. Moreover, it is not approved for the treatment of polymyalgia rheumatica (PMR) in Russia, which would render its use off-label for this indication. These factors underscore the relevance of evaluating alternative IL-6 inhibitors, such as olokizumab, in PMR patients requiring steroid-sparing therapy.

As noted before, tocilizumab was approved for the management of GCA a long time ago (GiActa study) (5), since then its use was endorsed by a number of clinical guidelines (6, 7) and is now used in Russian clinical practice (8). However, clinicians consider it to be effective in PMR as well, as confirmed by a number of real-life observations and studies (9–11). Nonetheless, tocilizumab has not been approved in PMR.

Giant cell arteritis and PMR are known to frequently overlap (with 40%–60% of patients with GCA reporting symptoms of PMR and 15%–20% of patients with PMR presenting with concurrent vasculitis or it develops in the future), have similar pathogenic mechanisms involving the interleukin 6 (IL-6) axis, affect similar age groups, and respond readily to treatment with CSs (12).

To summarize, GCA and PMR may be considered closely related medical conditions that require similar management (not in terms of treatment regimens but rather in terms of drug agents, primarily steroid-sparing) on the one hand, whereas some medications acting on the IL-6 axis may be regarded potentially effective in patients with these disorders.

The objective of this study is to evaluate clinical data on OKZ in patients with PMR with contraindications or inefficacy of methotrexate who needed steroid-sparing treatments. The long-term CS treatment in these patients was associated with a high risk of complications. Here we summarized data on patients with PMR, taking into account its greater prevalence in comparison with GCA.

Interleukin 6 is a pleiotropic pro-inflammatory cytokine (13–15) that plays a major role in the development of rheumatoid arthritis (RA) and other immune-mediated inflammatory rheumatological diseases. IL-6 inhibitors demonstrate marked anti-inflammatory activity, which is important in the management of conditions associated with an acute-phase inflammatory reaction such as GCA and PMR.

Olokizumab is a humanized monoclonal antibody of the immunoglobulin G4/kappa isotype (16) that selectively binds to human IL-6, effectively neutralizing the effects of Il-6 *in vivo* and *in vitro*. OKZ was studied in a series of multicenter, international, phase 3 trials that showed its safety and efficacy in over 2,400 patients with rheumatoid arthritis (17, 18).

## Materials and methods

Below we present a case series of OKZ use in patients with PMR. Although some patients also had a history of comorbid GCA, patients with evidence of active GCA were not included in our study for safety reasons. All patients were diagnosed with the condition in line with the EULAR/ACR 2012 classification criteria (19). For differential diagnosis, all patients completed a comprehensive evaluation including oncological screening and the exclusion of other rheumatological disorders and infections, with no data suggestive of the listed conditions identified. The patients were followed at 7 sites in Russia and had been informed about their treatment with OKZ for indications not listed in the instruction for use (off-label) and had signed the informed consent form before treatment initiation.

Methotrexate (MTX) was considered as a first-line steroid-sparing agent in all patients. However, its use was not feasible for the following reasons: contraindications [primarily decreased renal function with estimated glomerular filtration rate (eGFR) below 30 mL/min/1.73 m^2^, or liver abnormalities] were present in 5 out of 23 patients (21.7%); poor tolerability or patient refusal was documented in 6 cases (26.1%); and inadequate efficacy was observed in 12 patients (52.2%). In cases where MTX was used, the dose ranged from 7.5 to 15 mg per week, which is standard for PMR. Despite this, these patients either did not achieve disease control or were unable to reduce glucocorticoid doses without flare. Thus, all 23 patients had justified reasons for not receiving or discontinuing MTX and required an alternative steroid-sparing therapy. During the treatment process, planned therapy for concomitant diseases was continued. No new disease-modifying antirheumatic drugs were prescribed for the treatment of polymyalgia rheumatica, and the dose of existing ones was not increased. Symptomatic use of NSAIDs and adjustment of the GC dose were allowed (however, no increase in the GC dose was required). OKZ was used as subcutaneous injections 64 mg once every 4 weeks in all patients.

Efficacy evaluation was based on levels of acute-phase reactants (ESR and CRP), dynamics of Polymyalgia rheumatica-activity score (PMR-AS), and changes of glucocorticoid (GC) doses during OKZ treatment. All the parameters were assessed at baseline and weeks 4 and 12–24.

### Statistics

Results were presented using descriptive statistics for each time point. Continuous measures are reported as median with first and third quartiles to prevent potential distortion from non-normal distribution due to the small patient sample size. Categorical data are described using absolute values and percentages. 95% confidence intervals (CIs) for percentages were calculated using the Clopper-Pearson method.

For continuous variables, group comparisons were performed using the Mann-Whitney U test.

When comparing three or more dependent groups, the non-parametric Friedman test was used. *Post hoc* comparisons were conducted with the Conover-Iman test using the Holm correction.

To evaluate the discriminatory ability of quantitative features in predicting specific outcomes, ROC curve analysis and area under the curve (AUC) with 95% CI were applied. Cut-off values for quantitative features were determined based on the highest Youden’s index.

Linear regression was used to assess the predictive capability of the PMR-AS index model using explanatory variables selected through correlation analysis. Logistic regression was applied to evaluate the model’s predictive capacity for achieving low PMR activity (based on PMR-AS) and steroid-free remission.

Patients with PMR were considered to be in remission or low activity in case of PMR-AS values < 10, disease activity was moderate with PMR-AS ≥ 10 and ≤20, and PMR-AS activity > 20 was considered high.

For each endpoint and time point, the number of patients with available data (n) is reported in the corresponding tables/figures and text. No imputation of missing data was performed. All available data were included in the analysis. Patients with missing data at specific time points were excluded from those analyses.

All data provided as medians and IQR. All statistical tests were two-sided, with *p* < 0.050 considered statistically significant.

Analysis was performed using StatTech v. 4.7.3 (developer: StatTech LLC, Russia).

## Results

The study included 23 patients (Table 1). The age was 76.0 (72.0–81.0), ranging from 59 to 86 years. The disease duration at the start of treatment was 20.00 (5.50–42.50) months. The total OKZ treatment duration was 7.00 (6.00–10.50) months, with a minimum treatment duration of 3 months and maximum of 30 months.

**TABLE 1:** Descriptive statistics of quantitative variables at baseline.

Parameter*	Value
Age, years	76.00 (72.00–81.00)
Female, %	69.57
Number of patients, *n*	23
Disease duration at the start of treatment, months	20.00 (5.50–42.50)
Total OKZ treatment duration, months	7.00 (6.00–10.50)
ESR, mm/h	32.00 (20.00–47.00)
CRP, mg/l	14.10 (8.10–40.80)
PMR-AS, score	18.60 (14.20–24.44)
Steroid dose at OKZ initiation, mg/day	7.50 (5.00–15.00)

*Shown as Me (Q1–Q3), unless indicated otherwise.

The assessment was performed not at a single specific week, but over the period from Week 12 to Week 24. This approach was necessary because patients were enrolled across multiple centers, and strict adherence to a fixed visit schedule was not always feasible. Additionally, given the retrospective nature of the study, visit windows were variable. To ensure consistency in analysis and simplify the statistical processing, the data from this interval were pooled and analyzed as a single time point.

**FIGURE 1:**
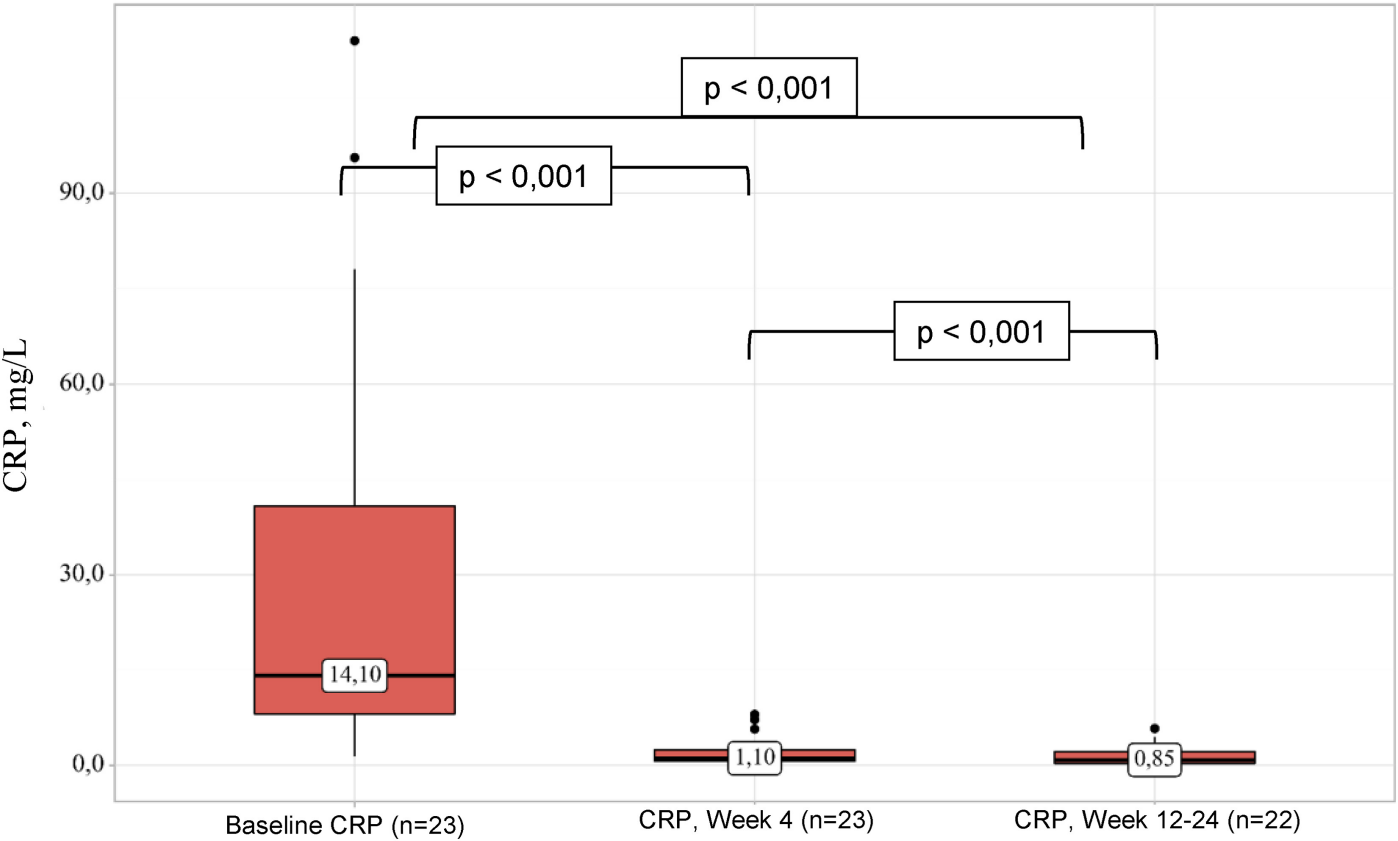
C-reactive protein (CRP) changes analysis.

The evaluation of laboratory parameters included analysis of ESR (Table 2) and of CRP (Figure 1). Both decreased during OKZ therapy. The baseline ESR value (Westergren method) was 32.0 (20.0–47.0) mm/h, while at Week 4 of treatment the median ESR decreased to 8.00 (6.00–13.00) mm/h. At Weeks 12–24 of treatment, the median ESR was 7.00 (5.00–8.50) mm/h. A similar trend was observed for CRP: the baseline value was 14.10 (8.10–40.80) mg/L, at Week 4 the median decreased to 1.10 (0.66–2.45) mg/L, and at Weeks 12–24 to 0.85 (0.30–2.15) mg/L. Both changes were statistically significant (*p* < 0.001; Friedman test).

**TABLE 2:** Erythrocyte sedimentation rate (ESR) changes.

Study timepoints						*p*
Baseline ESR		ESR, Week 4		ESR, Week 12–24		
Me	Q_1_–Q_3_	Me	Q_1_–Q_3_	Me	Q_1_–Q_3_	
32.00 (*n* = 23)	20.00–47.00	8.00 (*n* = 23)	6.00–13.00	7.00 (*n* = 23)	5.00–8.50	<0.001* *p* (*BaselineESRvs*.*Week* 4*ESR*) < 0.001 *p* (*BaselineESRvs*.*Week* 12_–24 ESR)_ < 0.001

*The differences between the values were statistically significant (*p* < 0.05).

**FIGURE 2:**
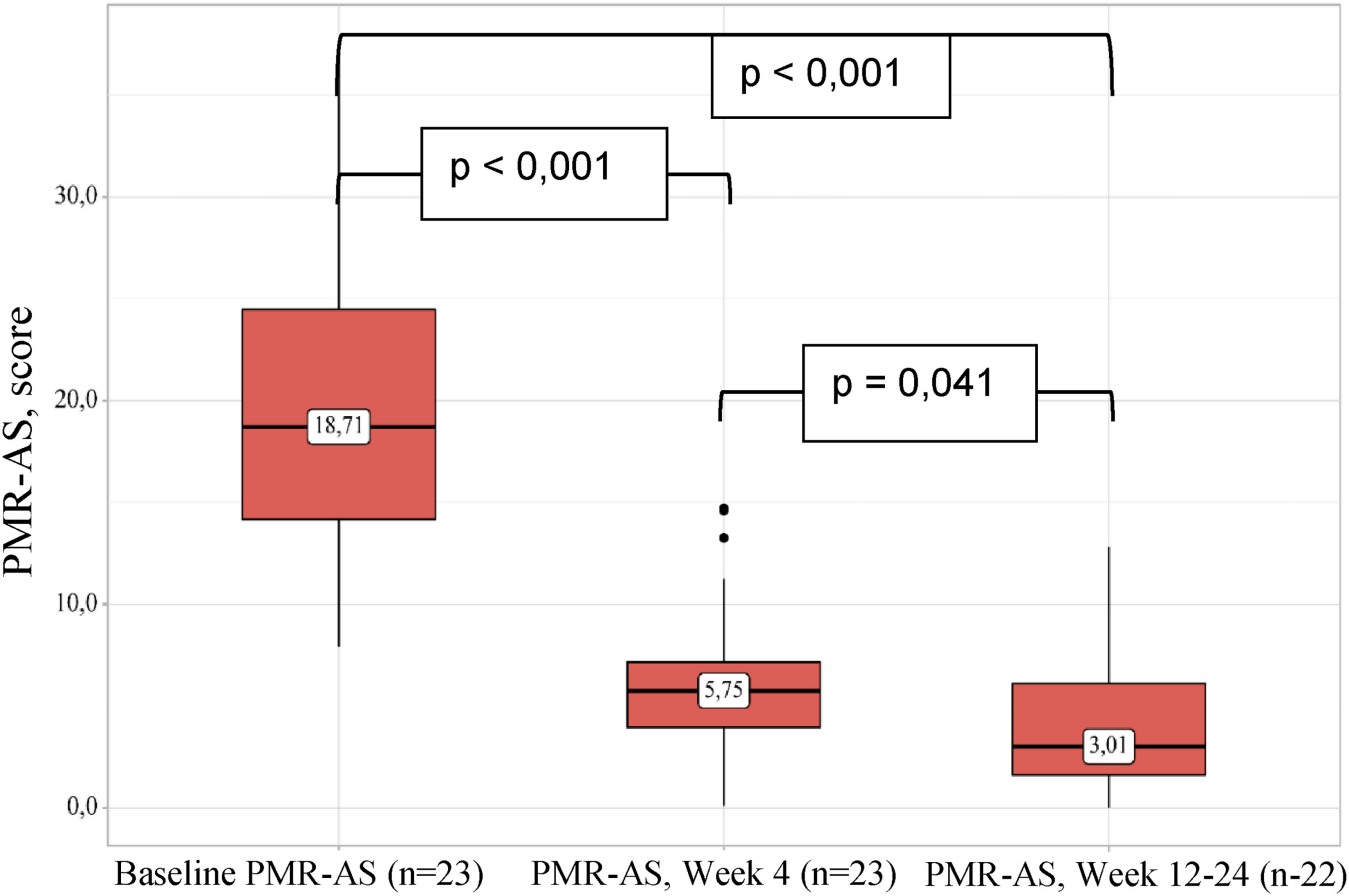
Polymyalgia rheumatica activity score (PMR-AS) changes analysis.

Polymyalgia rheumatica-activity score also decreased during treatment (Figure 2). PMR-AS is calculated as CRP (mg/dl) + VAS p (0–10) + VAS ph (0–10) + (MST (min) × 0.1) + EUL (3–0), where MST is the morning stiffness in minutes, and EUL is the ability to elevate the upper limbs. The baseline PMR-AS value was 18.6 (14.2–24.4) points, with the median decreasing to 5.40 (3.99–7.12) points at Week 4 of treatment. At Weeks 12–24, the median PMR-AS was 3.01 (1.62–6.09) points, *p* < 0.001. This may be consistent with a decrease in disease activity due to treatment.

**FIGURE 3:**
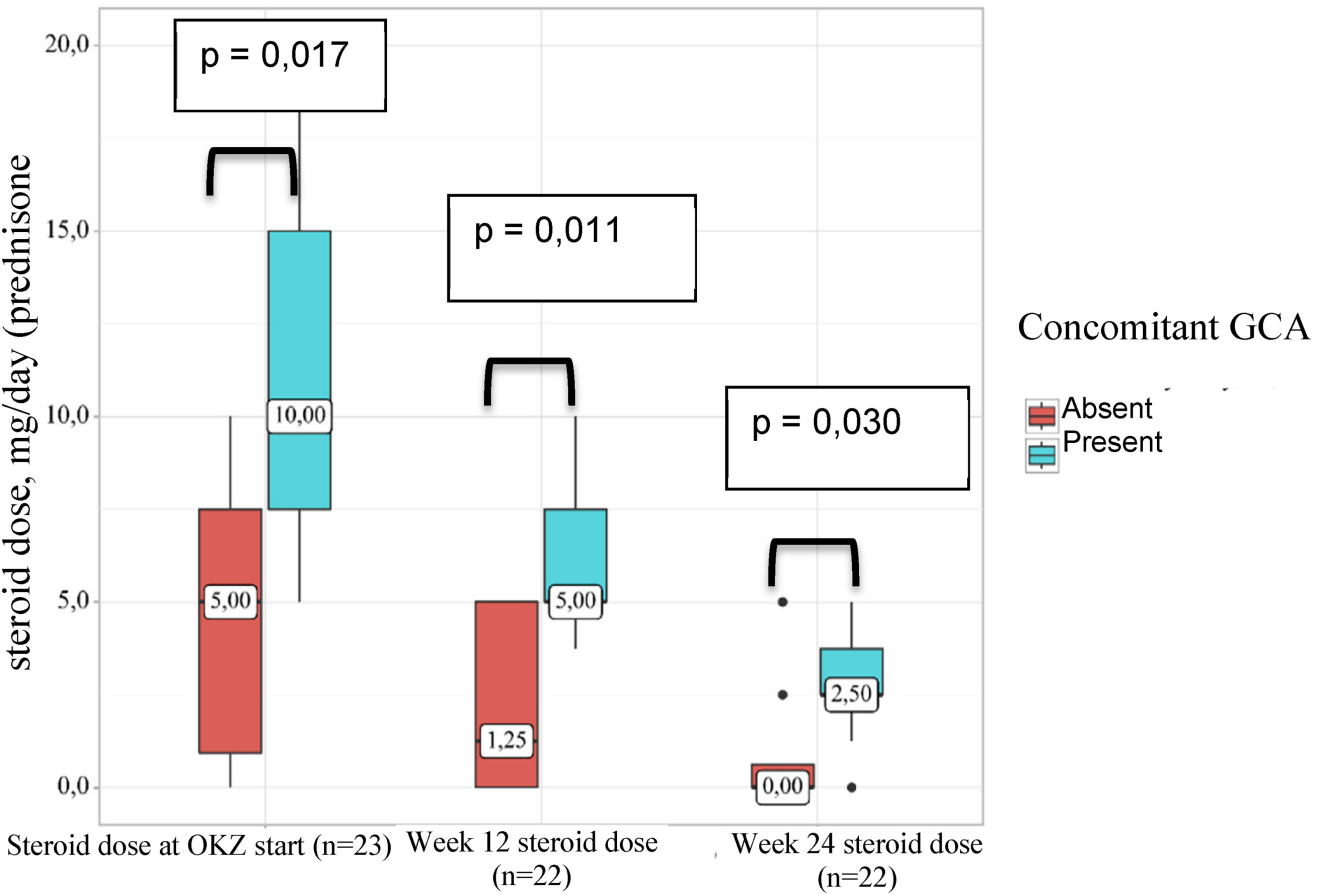
Analysis of changes in steroid dose depending on the presence of concomitant GCA.

**FIGURE 4:**
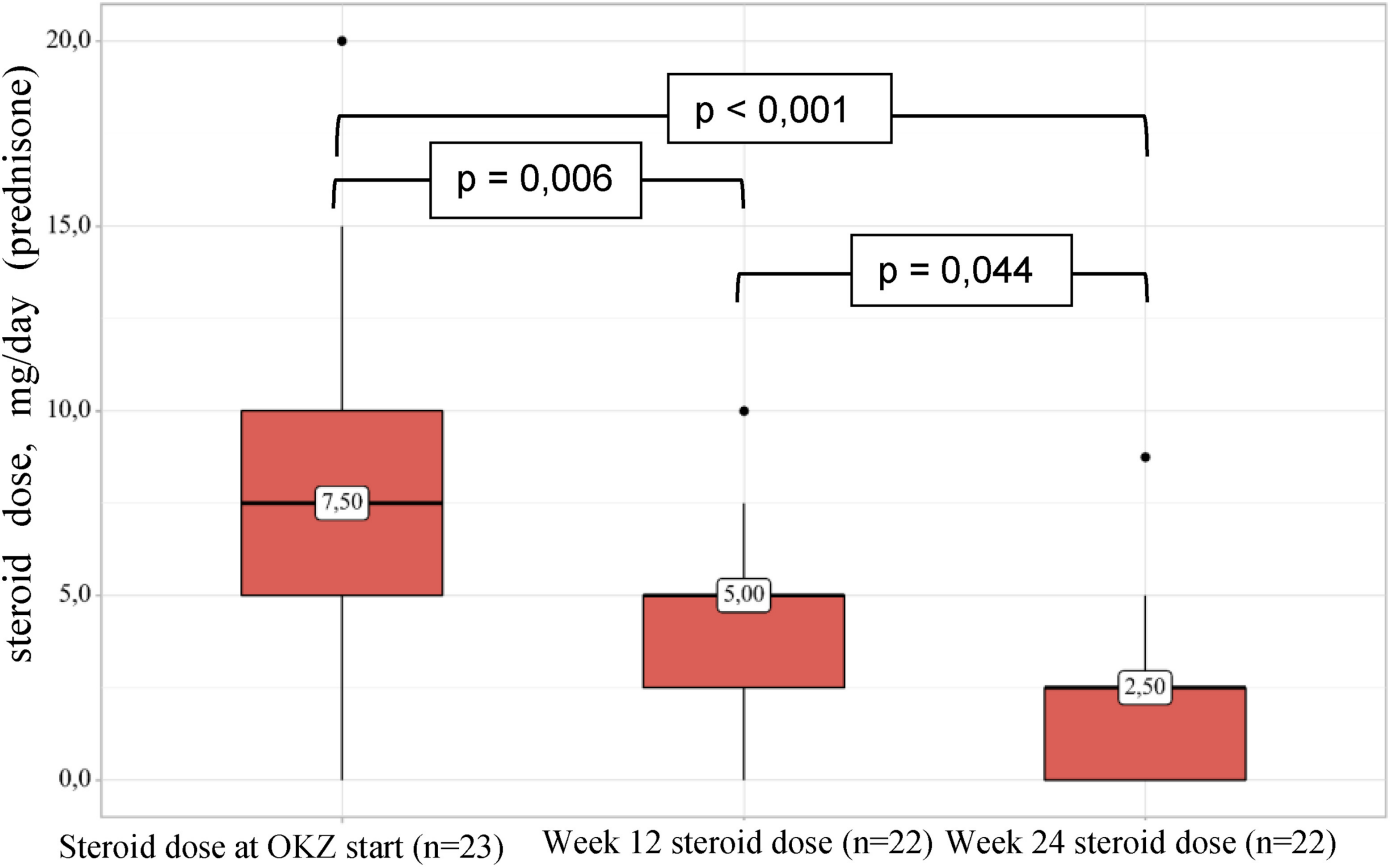
Analysis of steroid dose changes.

Glucocorticoid doses were also reduced during treatment (Figures 3, 4). At therapy initiation, the steroid dose was 7.5 (5.0–15.0) mg/day (prednisone equivalent). By week 12, the median steroid dose decreased to 5.00 (1.25–5.00) mg/day, and by week 24 to 2.50 (0.00–2.50) mg/day.

### Treatment outcome prediction

The following factors were considered as potential predictors of treatment success: patient age, disease duration, duration of OKZ therapy, presence of concomitant giant cell arteritis (GCA), baseline ESR and CRP levels. Positive treatment outcomes were defined as achieving low disease activity according to the PMR-AS index and achieving steroid-free remission.

1. Achieving steroid-free remission

9 out of 23 patients (39.1%) achieved steroid-free remission. The initial daily steroid dose showed significant association with the likelihood of achieving steroid-free remission in PMR. This intuitive finding did not warrant further predictive modeling. The dose of GCs was reduced after achieving clinical and laboratory remission at a rate not exceeding 1.25 mg per month in prednisolone equivalent (Figure 4). If signs of disease activity or manifestations of adrenal insufficiency appeared, no further reduction of GCs was carried out until the symptoms of adrenal insufficiency or signs of disease activity resolved. An increase in the GC dose was permitted within the framework of this study, but was not required for the patients we observed. Due to the fixed steroid tapering protocol, patients receiving higher initial doses were unable to completely discontinue steroids within the observation period, regardless of achieved control of immunoinflammatory activity.

No significant predictive value for achieving steroid-free remission was found for patient age, disease duration, duration of OKZ therapy, presence of concomitant GCA, baseline disease activity (including ESR and CRP levels).

1. Achievement of low disease activity in PMR

An analysis was conducted to identify predictors of achieving low disease activity in patients with PMR. It was found that disease duration at treatment initiation was a statistically significant predictor of disease activity at weeks 12–24 (AUC = 0.912; 95% CI: 0.683–1.000; *p* = 0.024), and hence a predictor of treatment outcome. The cut-off value of disease duration at treatment initiation that corresponded to the highest Youden index was 36.0. Low disease activity was predicted when the disease duration at the start of therapy was less than 36 months (3 years). The sensitivity and specificity of the resulting predictive model were 73.7% and 100.0%, respectively.

The presence of concomitant GCA, as well as baseline ESR and CRP levels, did not significantly affect the likelihood of achieving low disease activity. Additionally, no association was found between the presence of GCA and ESR or CRP values. However, it was noted that patients in the PMR + GCA group received higher doses of steroids (GC), and this difference was statistically significant. The data are presented in Figure 3.

A linear regression analysis was performed to evaluate the association between PMR-AS values at the end of follow-up (weeks 12–24) and various potential predictors: patient age, disease duration at the start of therapy, total duration of OKZ therapy, presence of concomitant GCA, baseline ESR, ESR at week 4, ESR at weeks 12–24, baseline CRP, CRP at week 4, and CRP at weeks 12–24. The number of observations at week 24 was 22/23. Data for the final time point were unavailable for one of the 23 patients, as this individual was enrolled at a later stage and had not completed the scheduled visit by the data cut-off date for the analysis. A significant association was found between PMR-AS at weeks 12–24 and CRP levels at week 4 and weeks 12–24. However, this relationship is of limited clinical value, as it reflects the mathematical contribution of CRP to the PMR-AS index and does not allow for reliable prediction of treatment response.

## Discussion

The findings are consistent with global practice, where higher doses of steroids are used in the treatment of GCA due to the associated risk of ischemic complications (in comparison to isolated PMR). However, this also necessitates increased clinical vigilance in this patient population, as GC-related adverse events are dose-dependent.

### Cohort analysis

The characteristics of the studied cohort were representative of the general PMR population. The median age was 76.0 (72.0–81.0) years, consistent with global data (20). A disease duration of about 2 years also appears sufficient for the identification of a cohort of patients who are difficult to treat with standard therapy (21). When steroid therapy is continued for more than 1–1.5 years, it often becomes clear that some patients cannot achieve dose tapering or discontinuation–so-called steroid dependency–which typically prompts the use of steroid-sparing agents. Given the relatively rapid onset of action described for IL-6 receptor inhibitors and OKZ in the literature–where the initial response is typically achieved within 1–2 months of treatment (4, 22, 23), –a 6-months treatment period appears sufficient to evaluate drug efficacy and its steroid-sparing potential. Patients with concomitant GCA received significantly higher steroid doses than those with isolated PMR, which is consistent with current treatment protocols that require higher steroid doses for GCA (24).

### Changes in parameters and prediction of outcomes of treatment with OKZ

Olokizumab treatment efficacy evaluation was based on using both laboratory (ESR, CRP) and composite indices, which included clinical manifestations and subjective assessments of disease activity (PMR-AS index). From a clinical standpoint, the most critical goal of therapy–and arguably the only meaningful treatment target in patients with low PMR activity–is steroid-sparing. Therefore, steroid dosage at treatment initiation and at the end of follow-up was also used as an endpoint in this analysis. This endpoint was used in several clinical trials evaluating agents acting on the IL-6 axis (e.g., sarilumab) (4) and is a reliable reflection of treatment success.

All assessed parameters showed statistically significant positive trends. As early as Week 4, CRP levels had dramatically decreased from 14.1 (8.10–40.80) to 1.10 (0.66–2.45), and this effect was maintained throughout follow-up. A similar trend was observed for ESR, which also declined markedly within the first month of therapy. These results are consistent with previous findings on the use of OKZ in rheumatoid arthritis, where rapid CRP reduction was also observed (17, 25). However, under IL-6 blockade, hepatic synthesis of acute-phase reactants is directly suppressed, leading to rapid normalization of CRP irrespective of the underlying inflammatory activity; therefore, it may underestimate residual disease activity in PMR, which is particularly relevant for PMR-AS, as CRP is embedded in its calculation., For this reason, ESR is considered an alternative marker during therapy with this drug class – and indeed, it followed a similar trend in our study.

That said, prior studies assessing PMR activity indices have demonstrated a very strong correlation between these two markers, suggesting their general interchangeability (26).

It is important to highlight that OKZ therapy not only resulted in favorable laboratory changes but also led to a significant clinical improvement, as reflected in the decrease in PMR-AS activity index. A marked reduction in PMR-AS activity index was observed between weeks 0 and 4 and was maintained through weeks 12–24. By the end of the follow-up period, none of the patients had high PMR activity, and only 3 of 22 individuals had moderate activity; the remainder [86.3% (19 of 22)] achieved low disease activity or remission.

Notably, the most pronounced improvement in disease activity was observed during the first month of therapy. This suggests that the clinical response to OKZ can be expected within the first 1–2 months, even in routine clinical practice. It is also important to note that the total duration of OKZ therapy was not a statistically significant predictor of achieving low disease activity (AUC = 0.536; 95% CI: 0.101–0.970; *p* = 0.869), which allows for a cautious conclusion: from a clinical standpoint, it may not be reasonable to continue treatment if no meaningful response is observed within the first 1–2 months.

On the contrary, disease duration at therapy initiation was shown to be a statistically significant predictor of activity at Weeks 12–24 (AUC = 0.912; 95% CI: 0.683–1.000; *p* = 0.024), and thus of treatment outcome. Thus, in practice, the earlier OKZ therapy is initiated (optimally within 3 years of starting steroid treatment, based on the obtained data), the higher the patient’s likelihood of achieving low PMR activity. These findings support the clinical rationale for earlier OKZ initiation, potentially after the first or second flare, as treatment efficacy may decline with longer disease duration. This conclusion is consistent with the evolving concept of early access to targeted therapies (27). Early intervention is particularly important in the treatment of conditions such as PMR, GCA, and systemic autoimmune diseases, where the therapeutic goal is not only to control disease complications within a “window of opportunity,” but also to prevent steroid-related adverse events. This trend mirrors current approaches to ANCA-associated vasculitis, where rapid tapering of steroids is prioritized through the use of steroid-sparing agents (28, 29). Thus, our findings suggest the potential clinical benefit of initiating OKZ earlier in the course of PMR.

However, the limitations of this study include its open-label design and relatively small sample size. Because no imputation was performed, results are based on complete-case analyses and may be subject to missing-data bias if patients with incomplete follow-up differed systematically from those with complete data. In this case series, loss to follow-up was limited (just one patient data was missed at Weeks 12–24). These results will require confirmation in randomized controlled trials (RCTs), which have already been initiated (30, 31). Additionally, our study did not include a longitudinal assessment of quality of life in PMR patients treated with OKZ, which is planned for future RCTs.

Preliminary results demonstrate that OKZ therapy can rapidly and significantly reduce PMR disease activity. The steroid-sparing effect of the drug was also evident: all patients achieved a substantial reduction in steroid doses, and many were able to discontinue steroids entirely. Our findings also indicate the potential for using OKZ as monotherapy without concomitant steroid use, achieving complete disease remission.

No patients experienced adverse events of moderate or severe intensity. Among the class-specific effects, mild elevations in liver transaminases and a decrease in leukocyte count were observed. However, none of these cases reached clinical significance or required treatment discontinuation. Respiratory infections were also reported, but there was no conclusive evidence linking them to the use of the study drug. A detailed analysis of adverse events was not within the scope of this study and was not performed, as the observed abnormalities did not affect treatment management.

The data we obtained are consistent with findings from larger clinical studies of other IL-6 pathway inhibitors in PMR (4, 9). Given the need for effective and safe control of PMR activity and the urgent requirement for steroid-sparing approaches in this disease, OKZ appears to be a promising therapeutic option. Its unique mechanism of action–direct IL-6 blockade (as opposed to receptor inhibition employed by tocilizumab and sarilumab)–may facilitate more pronounced effects on IL-6 trans-signaling compared to cis-signaling (32). This could potentially translate to superior efficacy and improved tolerability. These aspects will also require further investigation in controlled clinical trials. We should once again claim that this data must be interpreted as exploratory and hypothesis-generating due to the small sample size, the absence of external validation and the overall case-series based design.

## Data Availability

The raw data supporting the conclusions of this article will be made available by the authors, without undue reservation.
